# Group Home Staff Experiences With Work and Health in the COVID-19 Pandemic in Massachusetts

**DOI:** 10.1001/jamahealthforum.2023.0445

**Published:** 2023-04-07

**Authors:** Karen Donelan, Jessica Wolfe, Anna Wilson, Carie Michael, Cindy Chau, David Krane, Paula Silverman, Jessica E. Becker, David Cheng, Elizabeth Cella, Bruce Bird, Julie H. Levison, Brian G. Skotko, Stephen J. Bartels

**Affiliations:** 1 Mongan Institute, Massachusetts General Hospital, Harvard Medical School, Boston; 2 Department of Medicine, Massachusetts General Hospital, Harvard Medical School, Boston; 3 Heller School for Social Policy and Management, Brandeis University, Waltham, Massachusetts; 4 Vinfen Corporation, Cambridge, Massachusetts; 5 Division of Child and Adolescent Psychiatry, Department of Psychiatry, Massachusetts General Hospital, Boston; 6 Department of Psychiatry, Harvard Medical School, Boston, Massachusetts; 7 Biostatistics Center, Massachusetts General Hospital, Boston; 8 Kennedy Krieger Institute, Baltimore, Maryland; 9 Down Syndrome Program, Division of Medical Genetics and Metabolism, Department of Pediatrics, Massachusetts General Hospital, Boston; 10 Department of Pediatrics, Harvard Medical School, Boston, Massachusetts

## Abstract

**Importance:**

Direct reports of the experiences of staff working in group homes for people with serious mental illness (SMI) and/or intellectual or developmental disabilities (ID/DD) are rarely reported. Hearing from workers about their experiences in the COVID-19 pandemic may inform future workforce and public policy.

**Objective:**

To gather baseline data on worker experience with the perceived effects of COVID-19 on health and work in the pandemic prior to initiating an intervention to mitigate the spread of COVID-19 and to measure differences in worker experience by gender, race, ethnicity, education, and resident population served (persons with SMI and/or IDD/DD).

**Design, Setting, and Participants:**

This mixed-mode, cross-sectional survey study was conducted using online then paper-based self-administration from May to September 2021 at the end of the first year of the pandemic. Staff working in 415 group homes that provided care within 6 Massachusetts organizations serving adults aged 18 years or older with SMI and/or ID/DD were surveyed. The eligible survey population included a census of staff who were currently employed in participating group homes during the study period. A total of 1468 staff completed or partially completed surveys. The overall survey response rate was 44% (range by organization, 20% to 52%).

**Main Outcomes and Measures:**

Self-reported experiential outcomes were measured in work, health, and vaccine completion. Bivariate and multivariate analyses explore experiences by gender, race, ethnicity, education, trust in experts and employers, and population served.

**Results:**

The study population included 1468 group home staff (864 [58.9%] women; 818 [55.7%] non-Hispanic Black; 98 [6.7%] Hispanic or Latino). A total of 331 (22.5%) group home staff members reported very serious perceived effects on health; 438 (29.8%) reported very serious perceived effects on mental health; 471 (32.1%) reported very serious perceived effects on health of family and friends; and 414 reported very serious perceived effects (28.2%) on access to health services, with statistically significant differences observed by race and ethnicity. Vaccine acceptance was higher among persons with higher educational attainment and trust in scientific expertise and lower among persons who self-reported as Black or Hispanic/Latino. A total of 392 (26.7%) respondents reported needing support for health needs, and 290 (19.8%) respondents reported needing support for loneliness or isolation.

**Conclusions and Relevance:**

In this survey study, approximately one-third of group home workers reported serious personal health and access to health care barriers during the first year of the COVID-19 pandemic in Massachusetts. Addressing unmet health needs and access to health and mental health services, including inequities and disparities by race, ethnicity, and education, should benefit staff health and safety, as well as that of the individuals with disabilities who rely on them for support and care.

## Key Points

**Question:**

What are the experiences relating to work and health of staff working in group homes serving persons with serious mental illness and/or intellectual or developmental disability during the first year of the COVID-19 pandemic in Massachusetts?

**Findings:**

In this survey study of 1468 group home staff members, very serious perceived effects on health (22.5%), mental health (29.8%), health of family and friends (32.1%), and access to health services (28.2%) were reported. Black workers were more likely to report these adverse perceived effects and less likely to accept vaccination, as were people with less trust in experts and lower educational attainment.

**Meaning:**

Group home workers perceived their personal health and access to health care to be lower during their work in the first year of the COVID-19 pandemic in Massachusetts.

## Teaser

This survey study assesses the perceived effect of working during the COVID-19 pandemic on the personal health and access to health care of group home staff.

## Introduction

Since March 2020, frontline workers in the US have faced unprecedented challenges as the COVID-19 pandemic created upheaval around the globe. The pandemic has had negative outcomes on the lives of residents and staff of congregate care settings, especially in long-term care facilities for older adults,^1^ but also for group homes for individuals with serious mental illness (SMI) and/or intellectual or developmental disabilities (ID/DD). Group home workers experiences often are neglected in the public’s attention.^2^

Massachusetts experienced a sudden and dramatic emergence of COVID-19 in the spring of 2020. As of August 2022, there had been nearly 7000 deaths and nearly 43 000 cases of COVID-19 reported among residents of congregate care settings (nursing home, assisted living, rest home, independent living, and group homes) in Massachusetts.^3,4^ Moreover, the March 2020 public health emergency declaration profoundly transformed daily activities for those who lived and worked in these organizations by disrupting routines, forcing vulnerable people to quarantine, halting community work and recreation activities key to therapeutic programming, and isolating residents from friends and family. While policy changes since March 2020 have been frequent, in general, they have required that staff in congregate care facilities wear masks and practice other routine preventive measures.^5^ Group homes combine housing with services and therapeutic supports for individuals with SMI and/or ID/DD. Some residents have co-occurring medical complexity and functional impairments that may require substantial hands-on assistance. These homes are regulated by state agencies, including the Massachusetts Department of Developmental Services and the Massachusetts Department of Mental Health; staffing levels are mandated, and organizations must ensure minimum staffing requirements are met. There are presently more than 8000 residents living in homes overseen by Massachusetts Department of Developmental Services and more than 3000 in homes overseen by Massachusetts Department of Mental Health.^6^

Staff who work in group homes have been at particular risk of infection. Massachusetts has recorded nearly 3000 cases of COVID-19 among staff of congregate care settings (inclusive of group homes and nursing homes) and, as of August 2022, reported 37 known deaths among their staff.^3^ These data do not include the increased levels of physical and mental strain that group home staff experienced while under lockdown and quarantine with testing and personal protective equipment in short supply in the early surge.^7,8^ As some staff became ill and overwhelmed, some left jobs for other work or family responsibilities, while others moved into the group homes in which they worked to provide 24-hour supervision.^9^ In a 2021 national survey of 1414 people, nearly all US congregate care workers (96%) reported facing a staffing shortage and relied extensively on temporary, contract, and other workers.^10^

The congregate care workforce is racially and ethnically diverse, both nationally^11,12,13^ and throughout Massachusetts. The COVID-19 pandemic is known to have disproportionately affected frontline workers and people from underrepresented racial and ethnic backgrounds.^14,15,16^ In Massachusetts, non-Hispanic Black health care support workers had a mortality rate approximately 3 times higher than that of non-Hispanic White health care support workers.^17^ Organizational norms also include a more gender-diverse workforce than may be found in other care settings—gender concordance is common in homes that are generally gender-specific.

As part of a large-scale randomized effectiveness implementation study in Massachusetts examining the outcomes of different approaches to preventing COVID-19 in group homes for persons with SMI and/or ID/DD, we conducted a baseline survey of group home staff in 6 large organizations in Massachusetts 1 year after the onset of the pandemic. We report on group home staff experiences in the first year of the pandemic. This report is distinguished by focusing on the secondary outcomes of COVID-19 on residential care frontline workers for adults with SMI and ID/DD and spans multiple domains of work and health.

## Methods

### Study Design, Population, Data Collection, and Response

In this survey study, we conducted a mixed-mode survey of group home staff between May 1, 2021, and September 1, 2021. Eligible staff worked in 1 of 415 group homes that provide care within 6 Massachusetts organizations serving adults aged 18 years or older with SMI and/or ID/DD. These organizations were Vinfen, Bay Cove, Advocates, North Suffolk, Open Sky, and Riverside. Staff were initially eligible if their payroll records identified them as physically working in one of the homes as of January 2021; new staff hired thereafter could take the survey as well. The surveys were available in English, Spanish, and Haitian Creole, and support was provided to individuals who communicate verbally and nonverbally using American Sign Language.

Working with organizational partners, we conducted an iterative process of recruitment. We first distributed email invitations to staff with a link to a secure REDCap (Research Electronic Data Capture) questionnaire.^18,19^ A majority of staff had work email addresses on record, but initial survey response online was very limited. Factors limiting response to emailed surveys were reported to include (1) the January 2021 outreach list was dated; it contained 4614 names, but an average of only 3311 were actively working at any one time, (2) substantial employee turnover between January 2021 and survey launch in May 2021, and (3) limited internet access and limited devices for staff use during work hours. To reach staff without email addresses, including staff newly hired between January 2021 and May 2021, we hand-delivered or mailed paper packets to group homes to invite completion of the survey on paper. Finally, a member of the study staff reached out to group homes directly to schedule in-person or virtual meetings.

Staff who completed the survey received $10 gift cards. In accordance with institutional review board guidelines, organizations maintained individual information for paying incentives but had no access to responses of individuals; researchers accessed response data but had no access to individual identifiers. The research team accessed deidentified data only. Respondent identities were not stored with the data nor linkable with payroll or other protected health information; a separate form was used to gather data to pay the incentive without linking those identities to responses.

We used standards established by the American Association for Public Opinion Research (AAPOR)^20^ to calculate a response rate using the (All) tabulation and Response Rate 2 for multiple-mode surveys, attempting to account for the unique and changing circumstances of this pandemic effort, including persons of known and unknown eligibility, the possibility of group home–level refusal to distribute the survey to employees, and the eligibility criteria that a person was currently employed at the time of data collection.^20^ We used both complete and partially complete surveys in the numerator and a denominator that included known eligible, unknown ineligible, refusals, and used the household response to estimate noncontacts by individual staff. We did not adjust the response rates to include a proportion of unknown eligible in the rate numerator (see, for example, Response Rate 4).^20^

### Questionnaire Development

The questionnaire was developed for the purpose of this study and was informed by qualitative interviews conducted with 24 staff and 12 program directors who typically oversaw operations in multiple homes (see eMethods in Supplement 1). Content included information seeking and trust in information sources, the modified Coronavirus Impact Scale^21^ to measure the COVID-19 pandemic’s perceived effect on various areas of staff members’ work and personal lives, and access to health care and unmet needs items, including vaccine access. The questionnaire was designed to be administered in less than 10 minutes in any of multiple modes. Prior to administration, the survey tools underwent review by study team and stakeholder working group members; items were refined cognitive and pilot tests. Question and response text is shown in tables and figures.

Other measures included respondent characteristics, such as gender identity (man, woman, transgender, nonbinary, queer/genderqueer/nonbinary, questioning or unsure, something else, or prefer not to answer), ethnicity (Hispanic/Latino or not), and self-reported race (Asian or Asian American, Black or African American, More than 1 race, Native American, White, and other [unspecified]).

### Institutional Ethics Approval and Informed Consent

This study was approved by the Mass General Brigham institutional review board, the Massachusetts Department of Mental Health institutional review board, and the Massachusetts Department of Developmental Services research review committee. We obtained written informed consent from all survey participants prior to the administration of study surveys.

### Statistical Analysis

Survey responses, including respondent characteristics, were summarized overall, by group home division (ID/DD vs SMI), self-reported race and ethnicity, gender, and education. Pearson χ^2^ tests were used to test for statistically significant differences in respondent characteristics by home division and in levels of responses (very serious, somewhat serious, not serious, missing) by race and ethnicity, which were asked separately but combined for analysis (non-Hispanic Asian, Black (hereafter Black) vs non-Hispanic White (hereafter White), Hispanic vs White). The combined variable for race and ethnicity was limited to 3 categories (Black, Hispanic/Latino, and White) due to sample size considerations and the education variable was limited to 2 categories (less than college, college or more) for the same reason. In secondary analyses, multivariate logistic regression was used to test associations between health and work outcomes, including self-reported vaccination status controlling for gender, group home type, race and ethnicity, education, and trust in information sources. We used complete case analysis for the multivariate models. Item nonresponse is shown in all univariate and bivariate tables; cases with missing data or sample sizes inadequate for analyses were excluded from the multivariate models. Exclusions are described in the table footnotes. Analyses of data were performed using Stata statistical software, version 16 (StataCorp LLC). The 2-tailed statistical significance threshold was *P* < .05.

## Results

### Response

A total of 1468 staff completed or partially completed surveys. As reported in the Methods, a denominator of 3311 was used to calculate a response rate of 44% overall; the response rate varied by organization (46%, 52%, 20%, 33%, 35%, and 52%). A comparison of the respondents with the baseline demographic characteristics of all staff in the second quarter of 2021 indicates overrepresentation of Black employees (48% in baseline and 55.7% in survey responses) and underrepresentation of all other staff by race.

### Participant Characteristics

Table 1 shows characteristics of respondents for the total sample, and separately for staff working in groups homes for persons with ID/DD and SMI. Overall, 864 (58.9%) respondents were women, 818 (55.7%) were Black employees, 388 (26.4%) were White employees, and 98 (6.7%) were Hispanic or Latino employees. There are statistically significant differences observed in most demographic characteristics between workers in SMI and ID/DD group homes.

**Table 1.** Respondent Characteristics for Staff Working in Group Homes for People With Intellectual and Developmental Disabilities or Serious Mental Illness

Characteristic	No. (%)			% Difference (95% CI)^b^	*P* value
	All respondents (n = 1468)^a^	ID/DD staff (n = 639)	SMI staff (n = 730)		
**Vaccinated at time of survey**					
Yes	1132 (77.1)	471 (73.7)	584 (80.0)	6.3 (1.8 to 10.8)	.05
Not yet, but want to be	76 (5.2)	41 (6.4)	32 (4.4)	−2.0 (−4.4 to 0.4)	
No, and do not want to be	189 (12.9)	94 (14.7)	82 (11.2)	−3.5 (−7.1 to 0.1)	
Not answered/missing	71 (4.8)	33 (5.2)	32 (4.4)	−0.8 (−3.1 to 1.5)	
Gender identity					
Men	498 (33.9)	219 (34.3)	258 (35.3)	1.1 (−4.0 to 6.1)	.34
Women	864 (58.9)	379 (59.3)	418 (57.3)	−2.1 (−7.3 to 3.2)	
Transgender, nonbinary, genderqueer	21 (1.4)	NA^c^	NA^c^	1.0 (−0.1 to 2.1)	
Not answered/missing	85 (5.8)	37 (5.8)	42 (5.8)	0.0 (−2.5 to 2.4)	
Hispanic or Latino					
Yes	98 (6.7)	28 (4.4)	64 (8.8)	4.4 (1.8 to 7.0)	.005
No	1295 (88.2)	578 (90.5)	632 (86.6)	−3.9 (−7.2 to −0.5)	
Not answered/missing	75 (5.1)	33 (5.2)	34 (4.7)	−0.5 (−2.8 to 1.8)	
Race					
Asian	34 (2.3)	NA^c^	27 (3.7)	2.8 (1.2 to 4.3)	<.001
Black	818 (55.7)	422 (66.0)	352 (48.2)	−17.8 (−23.0 to −12.7)	
White	388 (26.4)	136 (21.3)	215 (29.5)	8.2 (3.6 to 12.8)	
Other non-Hispanic^d^	67 (4.6)	21 (3.3)	40 (5.5)	2.2 (0.0 to 4.3)	
Not answered/missing	63 (4.3)	26 (4.1)	32 (4.4)	0.3 (−1.8 to 2.4)	
Education					
High school graduate or some high school	210 (14.3)	118 (18.5)	83 (11.4)	−7.1 (−10.9 to −3.3)	<.001
Some college or technical/vocational school	464 (31.6)	214 (33.5)	222 (30.4)	−3.1 (−8.0 to 1.9)	
College graduate (BS, BA)	516 (35.1)	222 (34.7)	259 (35.5)	0.7 (−4.3 to 5.8)	
More than college	218 (14.9)	58 (9.1)	138 (18.9)	9.8 (6.2 to 13.4)	
Not answered/missing	60 (4.1)	27 (4.2)	28 (3.8)	−0.4 (−2.5 to 1.7)	
**Trust in information sources about COVID-19**					
Staff and directors in group home					
Yes, definitely	928 (63.2)	415 (64.9)	463 (63.4)	−1.5 (−6.6 to 3.6)	.77
Yes, somewhat	429 (29.2)	176 (27.5)	218 (29.9)	2.3 (−2.5 to 7.1)	
No	70 (4.8)	29 (4.5)	31 (4.2)	−0.3 (−2.5 to 1.9)	
Not answered/missing	41 (2.8)	19 (3.0)	18 (2.5)	−0.5 (−2.2 to 1.2)	
Experts in COVID-19 from hospitals or public health schools					
Yes, definitely	1050 (71.5)	469 (73.4)	514 (70.4)	−3.0 (−7.7 to 1.8)	.55
Yes, somewhat	304 (20.7)	122 (19.1)	159 (21.8)	2.7 (−1.6 to 7.0)	
No	71 (4.8)	29 (4.5)	38 (5.2)	0.7 (−1.6 to 2.9)	
Not answered/missing	43 (2.9)	19 (3.0)	19 (2.6)	−0.4 (−2.1 to 0.4)	

Abbreviations: ID/DD, intellectual disabilities/developmental disabilities; NA, not applicable; SMI, serious mental illness.

^a^ A total of 99 staff members cannot be allocated to a specific home and are thus included in “All Respondents” but not the ID/DD and SMI stratification.

^b^ Difference in proportion between ID/DD and SMI staff with 95% CIs by response category.

^c^ Per Human Research Protocol, cell sizes of 10 or less are not reported nor are data which would allow one to deduce or calculate them.

^d^ Non-Hispanic other includes Native American, more than 1 race, other (unspecified).

At the time of the survey, approximately 77% of all workers surveyed reported they had been vaccinated; 5% said they wanted to be vaccinated; 12.9% had been offered but did not want vaccination; and 5% did not answer the question. Vaccine acceptance was higher among White respondents as compared with Black or Hispanic respondents.

#### Perceived Effects of COVID-19

**Figure 1.**
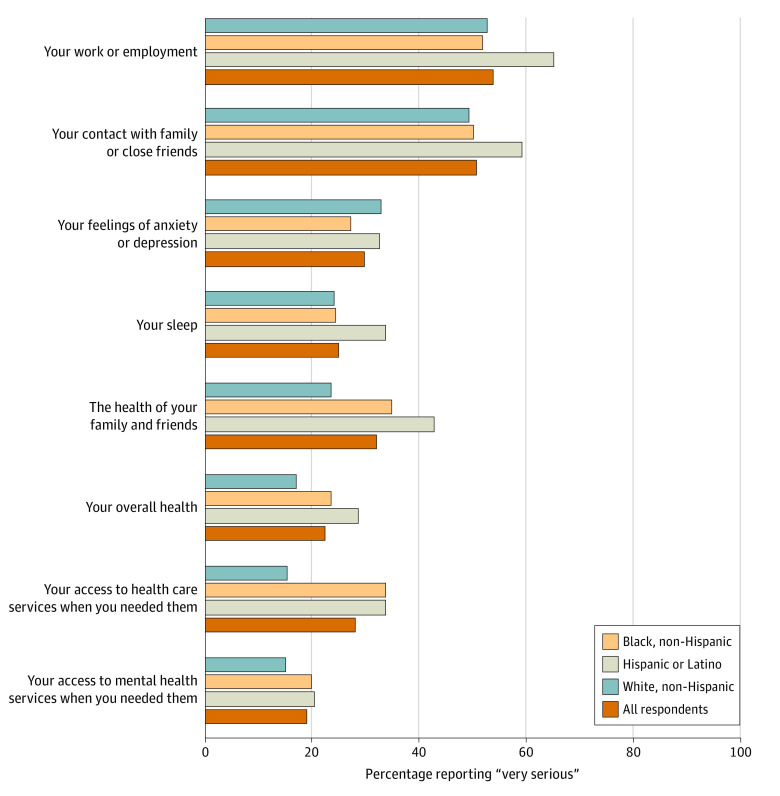
Group Home Workers’ Report of the Perceived COVID-19 Pandemic Effects The survey item was “In the past year, has the COVID-19 pandemic had a very serious, somewhat serious or not serious impact on...?”

Figure 1 (details in eTable 1 in Supplement 1) shows the responses of workers to a series of items on the perceived effects of COVID-19 on work and health. A majority of all workers reported “very serious” or “somewhat serious” effects in many aspects of their lives, including work, health, family, sleep, and other factors. More than 50% reported very serious effects on work or employment and on contact with family and friends, and 64.6% reported very serious or somewhat serious effects on access to health services. Figure 1 shows the very serious perceived effects reported by race and ethnicity in overall health (23.5% Black respondents, 28.6% Hispanic respondents, 17.0% White respondents) health of family and friends (34.8% Black respondents, 42.9% Hispanic respondents, 23.5% White respondents), work (52.0% Black respondents, 65.3% Hispanic respondents, 52.8% White respondents), access to health services (33.7% Black respondents, 33.7% Hispanic respondents, 15.5% White respondents), access to mental health services (19.9% Black respondents, 20.4% Hispanic respondents, 14.9% White respondents), and feelings of anxiety and depression (27.3% Black respondents, 32.7% Hispanic respondents, 33.0% White respondents). Given observed differences in educational attainment among workers in SMI and ID/DD homes, bivariate differences in these items were also examined by educational attainment level. College graduates were more likely than those with less than college education to report very serious perceived effects on their work (58.7% vs 48.2%); other differences were not statistically significant (see eTable 4 in Supplement 1); differences were not observed between employees in SMI and ID/DD homes.

#### Need for Support

**Figure 2.**
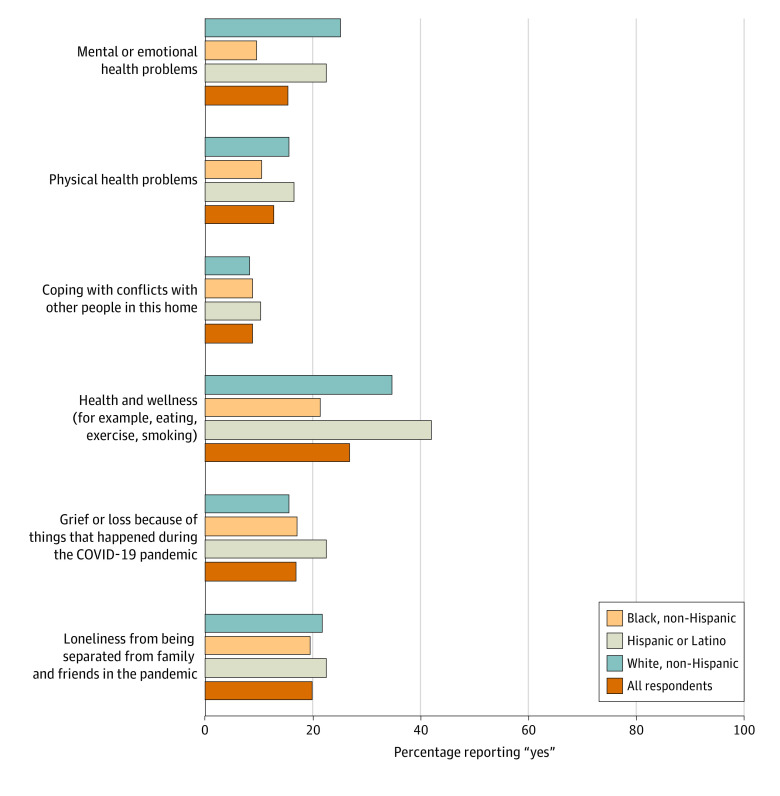
Group Home Workers’ Reported Need for Support The survey item was “Do you feel you need extra help right now with any of the following?”

Staff were also asked about areas in which they might need additional support (Figure 2; details in eTable 2 in Supplement 1). Approximately one-quarter (26.7%) of respondents reported needing support for health and wellness, 15.3% for mental health support, and 19.8% for loneliness or isolation. Statistically significant differences were observed by race in expressed need for support in the following areas: health and wellness (21.3% Black respondents, 41.8% Hispanic respondents, 34.5% White respondents), mental health (9.4% Black respondents, 22.4% Hispanic respondents, 25.0% White respondents), and physical health (10.4% Black respondents, 16.3% Hispanic respondents, 15.5% White respondents). Data for these items by worker education level revealed no statistically significant differences (eTable 5 in Supplement 1).

#### Trust in Leaders

**Figure 3.**
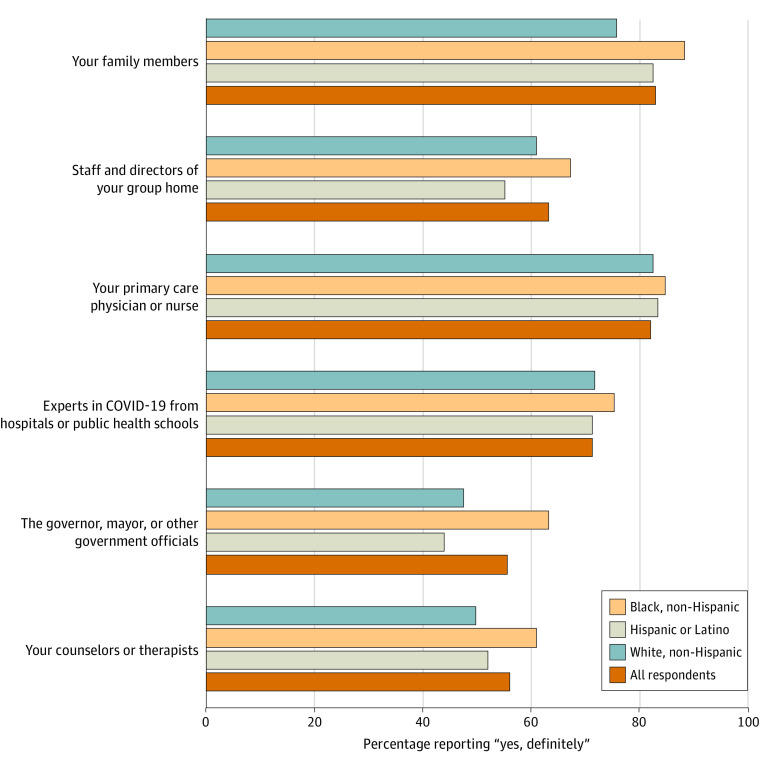
Group Home Workers’ Trust in Information Sources About COVID-19 The survey item was “For each item, indicate if you definitely or somewhat trust them to tell you the truth about COVID-19 or not.”

Group home workers were asked about their level of trust (“definitely yes,” “somewhat yes,” or “no”) with respect to a variety of leaders and health experts to “tell them the truth about COVID-19” (Figure 3; details in eTable 3 in Supplement 1). A total of 81.9% indicated that they definitely trusted primary care physicians or nurses, and 82.8% trusted family members. The highest proportion indicating no trust was 10.8% who did not trust the “governor, mayor or government officials,” but approximately 85% said they definitely or somewhat trusted these leaders. Workers who were Black were more likely to indicate trust in government than White workers (63.2% vs 47.7%). Workers with less than college education were more likely to express trust in family members (86.8% vs 82%) and in staff and directors of group homes (68.4% vs 60.6%) than were those with college education or higher (eTable 6 in Supplement 1).

### Multivariate Regression, Vaccine Completion

Table 2 shows results of logistic regression analysis of factors predicting vaccine completion and controlling for worker characteristics. Two model specifications were reported, a first controlling for race and ethnicity, educational attainment, and population in group homes (ID/DD, SMI), and where statistically significant differences were observed between workers by type of home. In the second model, we examine whether gender and trust in experts and employers as information sources were additional moderators of vaccine completion. In both models, work in ID/DD homes, educational attainment at less than college level, and identifying as a Black or Hispanic worker were associated with lower odds of having completed vaccination, although the CI in the full model does include one for work in an ID/DD home. Adding gender and trust to the full model shows that trust in experts increases.

**Table 2.** Logistic Regression Models of Summer 2021 Self-reported Vaccination Completion Among Group Home Staff^a^

Characteristic	Restricted model (n = 1217)^b^		Full model (n = 1195)	
	Odds ratio (95% CI)	*P* value	Odds ratio (95% CI)	*P* value
Group home population				
SMI	1 [Reference]	NA	1 [Reference]	NA
ID/DD	0.73 (0.54-0.98)	.03	0.75 (0.56-1.02)	.07
Race and ethnicity				
Black	0.64 (0.45-0.91)	.01	0.55 (0.38-0.81)	.002
Hispanic or Latino	0.42 (0.24-0.73)	.002	0.39 (0.22-0.70)	.001
White	1 [Reference]	NA	1 [Reference]	NA
Education				
College degree or more	1 [Reference]	NA	1 [Reference]	NA
Less than college	0.59 (0.44-0.79)	<.001	0.55 (0.41-0.75)	<.001
Gender				
Man	1 [Reference]	NA	1 [Reference]	NA
Woman	NA	NA	0.86 (0.62-1.19)	.37
Other	NA	NA	1.00 (0.20-4.95)	.99
Trust				
Trust staff leaders	NA	NA	0.89 (0.64-1.25)	.53
Trust experts	NA	NA	2.71 (1.93-3.80)	<.001
Constant	NA	NA	6.18 (3.7-10.3)	<.001

Abbreviations: ID/DD, Intellectual Disabilities/Developmental Disabilities; NA, not applicable; SMI, Serious Mental Illness.

^a^ Staff were asked, “Which of the following describes your experience with COVID-19 vaccines?” Unvaccinated staff includes those who answered, “I want to be vaccinated, but have not been vaccinated yet,” and, “I was offered the vaccine but did not want it.”

^b^ In the restricted model 63 respondents were excluded due to missing race and ethnicity; 101 as part of the Asian or Asian American, more than 1 race, Native American, and other (unspecified) race categories; 86 due to missing group home, and 1 due to missing educational attainment. For the full model, an additional 18 were excluded for missing sex, and 22 for missing trust variable responses.

## Discussion

In this survey study, the data reported here documented the profoundly challenging experiences of staff working at the front lines in group homes for persons with SMI and ID/DD in Massachusetts during the first year of the pandemic. To our knowledge, this study is the largest survey of the secondary outcomes of COVID-19 on residential care frontline workers for the challenging population of adults with disabilities and spans multiple domains of work and health. Here in Massachusetts, where the employed population is comprised of fewer than 10% Black workers, these group home data offer an unusual and compelling opportunity to have self-reported data from a workforce where the majority of workers are Black to explore self-reported data on health, work, and unmet needs. This representation feels especially important given the adverse outcomes of the pandemic on people of color and the relative paucity of survey data from frontline workers.

The majority of workers surveyed reported that the COVID-19 pandemic significantly affected their work, overall health, and mental health as well as impaired access to needed medical and mental health services. One in 7 workers reported needing extra help with loneliness, mental health problems, and health and wellness. The perceived effects of COVID-19 in this workforce varied significantly by race and ethnicity in almost every dimension that we measured.

Group homes are therapeutic environments that are critical to the care of vulnerable populations with disabilities that need extensive services and support. Residents of these environments may have multiple needs for physical health and social care. While in nonpandemic times many residents left their homes for work or day programs, these programs were largely discontinued during the pandemic. The responsibility to provide 24-hour care and support was largely assumed by staff, who in many cases left their own homes and worked overtime to respond to this crisis. The present findings documenting the negative outcomes of the pandemic on frontline workers, coupled with high rates of staff turnover and shortages, has substantial implications for a likely diminished capacity to address the complex needs of this highly vulnerable population of group home residents. In contrast to other congregate care facilities such as nursing homes, this workforce is more likely to be male. This difference likely results from the need for care professionals who are gender concordant with the resident population in group homes.

As group home staff faced increased workload demands and staffing shortages,^9^ they also experienced serious outcomes on their physical and mental health. The present findings are consistent with those of a national report about the direct support workforce. In that study, half of the staff reported physical and/or emotional burnout related to the COVID-19 pandemic.^22^ Other studies have also highlighted the psychological toll of the pandemic on staff, including increased work-related stress, fear of infection, and fear of transmission to family and friends.^23,24,25^ Despite these outcomes, not all staff who reported serious effect of COVID-19 also reported the need for additional support. This may indicate staff hesitancy to ask for support when needed; in offering interventions to support workers, it may be necessary to overcome this perception.

Direct support and other health care workers are at risk for burnout and other psychological consequences in their professional roles during emergent outbreaks of respiratory infections as in the SARS pandemic of the early 2000s.^11,26^ Understanding the differential effects of COVID-19 on the personal and professional lives of the workforce in congregate care settings is essential to assure that infection prevention strategies do not also cause detrimental and unintended harms. Knowing these consequences can allow for needed modifications to improve workplace environment to improve quality of life, such as staffing and task-shifting, and access to mental health and substance use support to sustain a healthy and productive workforce.^27^

These data were collected in a time frame 1 year into the pandemic as a first round of vaccines had been completed for many workers but before state policy makers and group home organizations had introduced mandates. Despite high rates of first vaccination in this staff cohort, these data do reveal some important issues for stakeholder employers, policy makers, and most especially for residents in group homes, notably that careful attention to educational and trust barriers may be needed to overcome initial resistance to vaccination and other infection control procedures among staff. Further, the physical and mental well-being of staff working in these conditions may require policies to assure workers are attending to their own health needs and have access to mental health supports.

## Limitations

This study has several limitations. The staff completing the survey while working in group homes in the Commonwealth of Massachusetts may not be generalizable to staff working in congregate care settings in other states. Surveys were distributed through employers; this may have influenced workers’ opportunity or willingness to participate in ways that we cannot measure. Response rates varied markedly across the 6 participating organizations; the effect of nonresponse in those settings is unknown. We deployed both web-based and paper surveys; due to privacy restrictions, we cannot eliminate the possibility that some people may have taken the survey in more than 1 mode. However, we estimate this to be no more than 1% of responses. The degree of staff turnover and employment of part-time and temporary staff made it difficult to establish the actual denominator of eligible employees during the study period and to report an accurate response rate. Based on demographic characteristics in payroll data, we may have underrepresented or overrepresented workers by race, ethnicity, gender, and education—missing data may account for some of these differences. We did not collect information on age or job tenure in the survey instrument and are unable to link survey responses to payroll data due to privacy restrictions and so could not control for these potentially important independent variables in this analysis.

## Conclusions

In this survey study, the assessment of a racially and ethnically diverse sample of 1468 staff working in Massachusetts group homes for persons with SMI and ID/DD confirms that this segment of the workforce has experienced substantial adverse outcomes because of the COVID-19 pandemic across multiple domains of physical, mental, and social health, yet they have also experienced substantial impaired access to needed services. Improving the working conditions of this workforce will benefit their health and safety, as well as that of the individuals with substantial disabilities and health challenges who rely on them for care.

## Notes

Supplement 1. **eMethods.** Survey **eTable 1.** Staff Reported Impacts of COVID-19 by Race and Ethnicity **eTable 2.** Staff Reported Needs for Support by Race and Ethnicity **eTable 3.** Staff Reported Trust in Sources of COVID-19 Information by Race and Ethnicity **eTable 4.** Staff Reported Impacts of COVID-19 by Education Level **eTable 5.** Staff Reported Needs for Support by Education Level **eTable 6**. Staff Reported Trust in Sources of COVID-19 Information by Education Level

Supplement 2.
Data sharing statement
