# Supplementary material for: Group Home Staff Experiences With Work and Health in the COVID-19 Pandemic in Massachusetts
Source: JAMA Health Forum. 2023 Apr 7;4(4):e230445. doi: 10.1001/jamahealthforum.2023.0445 (PMC10082407; doi:10.1001/jamahealthforum.2023.0445)
Supplement: Supplement 1. — eMethods. Survey eTable 1. Staff Reported Impacts of COVID-19 by Race and Ethnicity eTable 2. Staff Reported Needs for Support by Race and Ethnicity eTable 3. Staff Reported Trust in Sources of COVID-19 Information by Race and Ethnicity eTable 4. Staff Reported Impacts of COVID-19 by Education Level eTable 5. Staff Reported Needs for Support by Education Level eTable 6. Staff Reported Trust in Sources of COVID-19 Information by Education Level [file jamahealthforum-e230445-s001.pdf]

Supplemental Online Content

Donelan K, Wolfe J, Wilson A, et al. Group home staff experiences with work and health in the COVID-19 pandemic in Massachusetts. *JAMA Health Forum.* 2023;4(4):e230445.
doi:10.1001/jamahealthforum.2023.0445

eMethods. Survey

eTable 1. Staff Reported Impacts of COVID-19 by Race and Ethnicity

eTable 2. Staff Reported Needs for Support by Race and Ethnicity

eTable 3. Staff Reported Trust in Sources of COVID-19 Information by Race and Ethnicity

eTable 4. Staff Reported Impacts of COVID-19 by Education Level

eTable 5. Staff Reported Needs for Support by Education Level

eTable 6 . Staff Reported Trust in Sources of COVID-19 Information by Education Level

This supplemental material has been provided by the authors to give readers additional information about their work.

© 2023 Donelan K et al. *JAMA Health Forum.*

eMethods. Survey

COVID-19 NEEDS ASSESSMENT FOR GROUP HOME STAFF

Please use a blue or black pen, placing a check mark in the box next to the appropriate response as indicated: ☑.
In general, you should ***select only one answer*** *for each question or row.*

1. <u>In the past year,</u> has the COVID-19 pandemic had a very serious, somewhat serious or not serious impact on… (check one box on each row)

	Very serious	Somewhat Serious	Not serious
a. Your work or employment	☐	☐	☐
b. Your feelings of anxiety or depression	☐	☐	☐
c. Your access to health care services when you needed them	☐	☐	☐
d. Your access to mental health services when you needed them	☐	☐	☐
e. Your contact with family or close friends	☐	☐	☐
f. Your sleep	☐	☐	☐
g. Your overall health	☐	☐	☐
h. The health of your family and friends	☐	☐	☐

2. In the past month, how often did you… (check one box on each row)? If you work in more than one home, answer about the place you work most.

	Almost always	Sometimes	Almost never	Don't know
a. Wear a mask in this home when you were around other people?	☐	☐	☐	☐
b. Wash your hands or sanitize (eating, using the bathroom or touching others)?	☐	☐	☐	☐
c. Ask for help when you were worried or depressed?	☐	☐	☐	☐
d. Use social or physical distancing at this home (3-6 feet away from others)?	☐	☐	☐	☐
e. Tell staff if you had COVID symptoms (fever, cough, loss of smell or taste)	☐	☐	☐	☐
f. Worry that the people around you might give you COVID?	☐	☐	☐	☐
g. Feel that others in this home do not respect your opinions related to COVID?	☐	☐	☐	☐

3. For each item, indicate if you definitely or somewhat trust them to tell you the truth about COVID-19 or not.

	Yes, definitely	Yes, somewhat	No	N/A
a. Your family members	☐	☐	☐	
b. Staff and directors of your group home	☐	☐	☐	
c. Your primary care physician or nurse	☐	☐	☐	
d. Your counselors or therapists	☐	☐	☐	☐
e. Experts in COVID from hospitals or public health schools	☐	☐	☐	
f. The governor, mayor or other government officials	☐	☐	☐	

4. In general, how important is to you to get health information from … (check one box on each row)

	Very important	Somewhat important	Not important
a. People who are the same gender as you?	☐	☐	☐
b. People with the same racial or ethnic background as you?	☐	☐	☐
c. People with the same health care needs as you (e.g., a peer)?	☐	☐	☐

5. Do you feel that you need extra help right now with any of the following (check one box on each row)

	Yes	No	Don't Know
a. Mental or emotional health problems?	☐	☐	☐

© 2023 Donelan K et al. *JAMA Health Forum.*

b. Physical health problems?	☐	☐	☐
c. Finding a job?	☐	☐	☐
d. Coping with conflicts with other people in your home?	☐	☐	☐
e. Health and wellness? (for example eating, exercise, smoking)	☐	☐	☐
f. Grief or loss because of things that happened during the COVID pandemic?	☐	☐	☐
g. Loneliness from being separated from family and friends in the pandemic?	☐	☐	☐

6. **Which of the following describes your experience with COVID-19 vaccines?**
   - ☐ I have been vaccinated **(SKIP TO 7)**
   - ☐ I want to be vaccinated, but have not been vaccinated yet
   - ☐ I was offered the vaccine, but did not want it

   6a. Why have you not been vaccinated? (CHECK ALL THAT APPLY)
   - ☐ I wanted to see how other people reacted
   - ☐ My religion does not allow me to get a vaccine
   - ☐ My parent or guardian does not want me to get it
   - ☐ My doctor does not want me to get it
   - ☐ I am worried about side effects
   - ☐ I do not think I need it
   - ☐ I do not like shots
   - ☐ I do not trust the government recommendations
   - ☐ Some other reason (please explain)

7. **Do you know how to use computers, laptop or smartphones to look at information or communicate online?**
   - ☐ Yes, I can do this
   - ☐ Yes, but I need help to do this
   - ☐ No, I do not know how to do this

8. **When you learn new things, which ONE of the following do you like best?**
   - ☐ Reading books or brochures with pictures and diagrams
   - ☐ Watching videos on TV or computer (Youtube, Vimeo)
   - ☐ Learning from a teacher or other person to help me
   - ☐ Internet courses with quizzes to check my learning

9. **Which of the following describes your gender?**
   - ☐ Man
   - ☐ Woman
   - ☐ Transgender or nonbinary
   - ☐ Queer, genderqueer, or non-binary
   - ☐ Questioning or unsure
   - ☐ Something else (specify_______________)
   - ☐ Prefer not to answer

10. **Are you of Hispanic or Latino background, such as Mexican, Puerto Rican, Cuban, or other Latin American background? (check one)**
    - ☐ Yes
    - ☐ No

11. **Do you consider yourself to be…? (check one)**
    - ☐ White
    - ☐ Black or African-American
    - ☐ Asian or Asian-American

© 2023 Donelan K et al. *JAMA Health Forum.*

☐ Native American
☐ More than one race
☐ Other (please specify) ____________

12. **What is the last grade that you completed in school? (check one)**
☐ Less than high school
☐ High school graduate (grade 12 or GED)
☐ Some college or technical/vocational school
☐ College graduate (B.S., B.A.)
☐ More than college

© 2023 Donelan K et al. *JAMA Health Forum.*

eTable 1. Staff Reported Impacts of COVID-19 by Race and Ethnicity

	All Respondents (n=1468)[a]	Non-Hispanic White (n=388)	Non-Hispanic Black (n=818)				Hispanic or Latino (n=98)			
	No. (%)	No. (%)	No. (%)	% difference (95% CI)[b]	P-value (vs. Non-Hispanic White)		No. (%)	% difference (95% CI)[b]	P-value (vs. Non-Hispanic White)	
**In the past year, has the COVID-19 pandemic had a very serious, somewhat serious or not serious impact on…?**										
Your work or employment										
Very serious	792 (54.0)	205 (52.8)	425 (52.0)	-0.9% (-6.9 , 5.2)	0.775		64 (65.3)	12.5% (1.8 , 23.1)	0.026	*
Somewhat serious	473 (32.2)	138 (35.6)	259 (31.7)	-3.9% (-9.6 , 1.8)	0.178		26 (26.5)	-9.0% (-19.0 , 0.9)	0.091	
Not serious	176 (12.0)	40 (10.3)	114 (13.9)	3.6% (-0.2 , 7.5)	0.078		‡	-2.1% (-8.4 , 4.1)	0.525	
Missing	27 (1.8)	‡	20 (2.4)	1.2% (-0.4 , 2.7)	0.188		‡	-1.3% (-2.4 , -0.2)	0.259	
Your feelings of anxiety or depression										
Very serious	438 (29.8)	128 (33.0)	223 (27.3)	-5.7% (-11.3 , -0.1)	0.041	*	32 (32.7)	-0.3% (-10.7 , 10.1)	0.949	
Somewhat serious	559 (38.1)	168 (43.3)	290 (35.5)	-7.8% (-13.8 , -1.9)	0.009	*	39 (39.8)	-3.5% (-14.4 , 7.4)	0.531	
Not serious	446 (30.4)	89 (22.9)	286 (35.0)	12.0% (6.7 , 17.3)	0.000	*	26 (26.5)	3.6% (-6.1 , 13.3)	0.455	
Missing	25 (1.7)	‡	19 (2.3)	1.5% (0.2 , 2.9)	0.060		‡	0.2% (-1.9 , 2.4)	0.809	
Your access to health care services when you needed them										
Very serious	414 (28.2)	60 (15.5)	276 (33.7)	18.3% (13.4 , 23.1)	0.000	*	33 (33.7)	18.2% (8.2 , 28.2)	0.000	*
Somewhat serious	534 (36.4)	162 (41.8)	283 (34.6)	-7.2% (-13.0 , -1.3)	0.016	*	31 (31.6)	-10.1% (-20.6 , 0.3)	0.067	
Not serious	494 (33.7)	160 (41.2)	244 (29.8)	-11.4% (-17.2 , -5.6)	0.000	*	32 (32.7)	-8.6% (-19.1 , 1.9)	0.120	
Missing	26 (1.8)	‡	15 (1.8)	0.3% (-1.2 , 1.8)	0.722		‡	0.5% (-2.6 , 3.6)	0.731	
Your access to mental health services when you needed them										
Very serious	281 (19.1)	58 (14.9)	163 (19.9)	5.0% (0.5 , 9.5)	0.037	*	20 (20.4)	5.5% (-3.3 , 14.2)	0.188	
Somewhat serious	410 (27.9)	126 (32.5)	214 (26.2)	-6.3% (-11.9 , -0.8)	0.023	*	29 (29.6)	-2.9% (-13.1 , 7.3)	0.584	
Not serious	725 (49.4)	193 (49.7)	409 (50.0)	0.3% (-5.8 , 6.3)	0.933		47 (48.0)	-1.8% (-12.9 , 9.3)	0.752	
Missing	52 (3.5)	11 (2.8)	32 (3.9)	1.1% (-1.0 , 3.2)	0.346		‡	-0.8% (-4.0 , 2.5)	0.663	
Your contact with family or close friends										
Very serious	744 (50.7)	191 (49.2)	411 (50.2)	1.0% (-5.0 , 7.1)	0.741		58 (59.2)	10.0% (-1.0 , 20.9)	0.078	
Somewhat serious	420 (28.6)	127 (32.7)	228 (27.9)	-4.9% (-10.4 , 0.7)	0.084		26 (26.5)	-6.2% (-16.1 , 3.7)	0.238	
Not serious	278 (18.9)	67 (17.3)	163 (19.9)	2.7% (-2.0 , 7.3)	0.272		13 (13.3)	-4.0% (-11.7 , 3.7)	0.340	
Missing	26 (1.8)	‡	16 (2.0)	1.2% (-0.1 , 2.5)	0.123		‡	0.2% (-1.9 , 2.4)	0.809	
Your sleep										
Very serious	368 (25.1)	94 (24.2)	199 (24.3)	0.1% (-5.1 , 5.3)	0.970		33 (33.7)	9.4% (-0.8 , 19.7)	0.057	
Somewhat serious	525 (35.8)	142 (36.6)	286 (35.0)	-1.6% (-7.4 , 4.2)	0.579		40 (40.8)	4.2% (-6.6 , 15.1)	0.441	
Not serious	546 (37.2)	149 (38.4)	312 (38.1)	-0.3% (-6.1 , 5.6)	0.931		24 (24.5)	-13.9% (-23.7 , -4.1)	0.010	*
Missing	29 (2.0)	‡	21 (2.6)	1.8% (0.4 , 3.2)	0.037	*	‡	0.2% (-1.9 , 2.4)	0.809	
Your overall health										
Very serious	331 (22.5)	66 (17.0)	192 (23.5)	6.5% (1.7 , 11.2)	0.011	*	28 (28.6)	11.6% (1.9 , 21.3)	0.010	*
Somewhat serious	551 (37.5)	158 (40.7)	303 (37.0)	-3.7% (-9.6 , 2.2)	0.219		36 (36.7)	-4.0% (-14.7 , 6.7)	0.471	
Not serious	547 (37.3)	159 (41.0)	295 (36.1)	-4.9% (-10.8 , 1.0)	0.100		33 (33.7)	-7.3% (-17.9 , 3.3)	0.186	

© 2023 Donelan K et al. *JAMA Health Forum.*

Missing	39 (2.7)	‡	28 (3.4)	2.1% (0.5 , 3.8)	0.034	*	‡	-0.3% (-2.6 , 2.0)	0.830	
The health of your family and friends										
Very serious	471 (32.1)	91 (23.5)	285 (34.8)	11.4% (6.1 , 16.7)	0.000	*	42 (42.9)	19.4% (8.7 , 30.1)	0.000	*
Somewhat serious	620 (42.2)	204 (52.6)	312 (38.1)	-14.4% (-20.4 , -8.5)	0.000	*	40 (40.8)	-11.8% (-22.7 , -0.8)	0.037	*
Not serious	351 (23.9)	89 (22.9)	204 (24.9)	2.0% (-3.1 , 7.1)	0.449		15 (15.3)	-7.6% (-15.9 , 0.6)	0.100	
Missing	26 (1.8)	‡	17 (2.1)	1.0% (-0.4 , 2.4)	0.194		‡	0.0% (-2.2 , 2.2)	0.993	

[‡]Per Human Research Protocol, cell sizes of 10 or less are not reported nor are data which would allow one to deduce or calculate them. *indicates P-values < 0.05. [a]Non-Hispanic Asian and Non-Hispanic Other were excluded from this table due to small cell sizes; their responses are reflected in “All Respondents.” [b]Difference in proportion between the specified racial/ethnic group and non-Hispanic White with 95% confidence intervals, by response category.

© 2023 Donelan K et al. *JAMA Health Forum.*

eTable2. Staff Reported Needs for Support by Race and Ethnicity

	All Respondents (n=1468)[a]	Non-Hispanic White (n=388)	Non-Hispanic Black (n=818)				Hispanic or Latino (n=98)			
	No. (%)	No. (%)	No. (%)	% difference (95% CI)[b]	P-value (vs. Non-Hispanic White)		No. (%)	% difference (95% CI)[b]	P-value (vs. Non-Hispanic White)	
**Do you feel that you need extra help right now with any of the following…?**										
Mental or emotional health problems?										
Yes	225 (15.3)	97 (25.0)	77 (9.4)	-15.6% (-20.3 , -10.8)	0.000	*	22 (22.4)	-2.6% (-11.9 , 6.8)	0.600	
No	1109 (75.5)	272 (70.1)	690 (84.4)	14.2% (9.1 , 19.4)	0.000	*	71 (72.4)	2.3% (-7.6 , 12.3)	0.649	
Do not know	81 (5.5)	19 (4.9)	46 (5.6)	0.7% (-1.9 , 3.4)	0.602		‡	0.2% (-4.7 , 5.1)	0.933	
Missing	53 (3.6)	‡	‡	0.6% (0.1 , 1.1)	0.123		‡	0.0% (0.0 , 0.0)		
Physical health problems?										
Yes	187 (12.7)	60 (15.5)	85 (10.4)	-5.1% (-9.2 , -0.9)	0.011	*	16 (16.3)	0.9% (-7.3 , 9.0)	0.834	
No	1180 (80.4)	318 (82.0)	702 (85.8)	3.9% (-0.7 , 8.4)	0.083		80 (81.6)	-0.3% (-8.9 , 8.2)	0.940	
Do not know	44 (3.0)	‡	25 (3.1)	1.0% (-0.8 , 2.8)	0.323		‡	0.0% (-3.2 , 3.1)	0.990	
Missing	57 (3.9)	‡	‡	0.2% (-0.7 , 1.1)	0.663			-0.5% (-1.2 , 0.2)	0.476	
Coping with conflicts with other people in this home?										
Yes	129 (8.8)	32 (8.2)	71 (8.7)	0.4% (-2.9 , 3.8)	0.802		10 (10.2)	2.0% (-4.6 , 8.5)	0.538	
No	1233 (84.0)	341 (87.9)	712 (87.0)	-0.8% (-4.8 , 3.1)	0.680		85 (86.7)	-1.2% (-8.6 , 6.3)	0.757	
Do not know	45 (3.1)	11 (2.8)	27 (3.3)	0.5% (-1.6 , 2.5)	0.665		‡	0.2% (-3.6 , 4.0)	0.905	
Missing	61 (4.2)	‡	‡	-0.1% (-1.3 , 1.2)	0.931		‡	-1.0% (-2.0 , 0.0)	0.313	
Health and wellness? (for example eating, exercise, smoking)										
Yes	392 (26.7)	134 (34.5)	174 (21.3)	-13.3% (-18.8 , -7.8)	0.000	*	41 (41.8)	7.3% (-3.6 , 18.2)	0.179	
No	981 (66.8)	246 (63.4)	613 (74.9)	11.5% (5.9 , 17.2)	0.000	*	53 (54.1)	-9.3% (-20.3 , 1.6)	0.090	
Do not know	35 (2.4)	‡	25 (3.1)	1.8% (0.1 , 3.4)	0.066		‡	-0.3% (-2.6 , 2.0)	0.830	
Missing	60 (4.1)	‡	‡	0.0% (-1.1 , 1.0)	0.940		‡	2.3% (-1.2 , 5.8)	0.067	
Grief or loss because of things that happened during the COVID pandemic?										
Yes	247 (16.8)	60 (15.5)	139 (17.0)	1.5% (-2.9 , 6.0)	0.504		22 (22.4)	7.0% (-2.0 , 16.0)	0.099	
No	1095 (74.6)	314 (80.9)	630 (77.0)	-3.9% (-8.8 , 0.9)	0.124		72 (73.5)	-7.5% (-17.0 , 2.1)	0.103	
Do not know	67 (4.6)	14 (3.6)	42 (5.1)	1.5% (-0.9 , 3.9)	0.124		‡	-0.5% (-4.4 , 3.3)	0.792	
Missing	59 (4.0)	‡	‡	0.9% (0.2 , 1.5)	0.068		‡	1.0% (-1.0 , 3.0)	0.046	*
Loneliness from being separated from family and friends in the pandemic?										
Yes	290 (19.8)	84 (21.6)	159 (19.4)	-2.2% (-7.1 , 2.7)	0.371		22 (22.4)	0.8% (-8.4 , 10.0)	0.864	
No	1053 (71.7)	284 (73.2)	618 (75.6)	2.4% (-2.9 , 7.7)	0.379		70 (71.4)	-1.8% (-11.7 , 8.2)	0.725	
Do not know	59 (4.0)	16 (4.1)	30 (3.7)	-0.5% (-2.8 , 1.9)	0.699		‡	-1.1% (-5.0 , 2.9)	0.628	
Missing	66 (4.5)	‡	11 (1.3)	0.3% (-1.0 , 1.6)	0.646		‡	2.0% (-1.5 , 5.6)	0.132	

‡Per Human Research Protocol, cell sizes of 10 or less are not reported nor are data which would allow one to deduce or calculate them. *indicates P-values < 0.05. [a]Non-Hispanic Asian and Non-Hispanic Other were excluded from this table due to small cell sizes; their responses are reflected in "All Respondents." [b]Difference in proportion between the specified

© 2023 Donelan K et al. *JAMA Health Forum.*

racial/ethnic group and non-Hispanic White with 95% confidence intervals, by response category.

© 2023 Donelan K et al. *JAMA Health Forum.*

eTable3. Staff Reported Trust in Sources of COVID-19 Information by Race and Ethnicity

	All Respondents (n=1468)[a]	Non-Hispanic White (n=388)	Non-Hispanic Black (n=818)				Hispanic or Latino (n=98)			
	No. (%)	No. (%)	No. (%)	% difference (95% CI)[b]	P-value (vs. Non-Hispanic White)		No. (%)	% difference (95% CI)[b]	P-value (vs. Non-Hispanic White)	
**For each item, indicate if you definitely or somewhat trust them to tell you the truth about COVID-19 or not.**										
Your family members										
Yes, definitely	1216 (82.8)	294 (75.8)	722 (88.3)	12.5% (7.7 , 17.3)	0.000	*	81 (82.7)	6.9% (-1.7 , 15.5)	0.147	
Yes, somewhat	175 (11.9)	77 (19.8)	70 (8.6)	-11.3% (-15.7 , -6.9)	0.000	*	14 (14.3)	-5.6% (-13.5 , 2.4)	0.207	
No	45 (3.1)	16 (4.1)	21 (2.6)	-1.6% (-3.8 , 0.7)	0.143		‡	-1.1% (-5.0 , 2.9)	0.628	
Missing	32 (2.2)	‡	‡	0.4% (-0.4 , 1.1)	0.415		‡	-0.3% (-0.8 , 0.2)	0.615	
Staff and directors of your group home										
Yes, definitely	928 (63.2)	236 (60.8)	549 (67.1)	6.3% (0.5 , 12.1)	0.032	*	54 (55.1)	-5.7% (-16.7 , 5.3)	0.302	
Yes, somewhat	429 (29.2)	129 (33.3)	217 (26.5)	-6.7% (-12.3 , -1.1)	0.016	*	42 (42.9)	9.6% (-1.3 , 20.5)	0.075	
No	70 (4.8)	22 (5.7)	39 (4.8)	-0.9% (-3.6 , 1.8)	0.504		‡	-3.6% (-7.3 , 0.0)	0.138	
Missing	41 (2.8)	‡	13 (1.6)	1.3% (0.3 , 2.3)	0.044	*	‡	-0.3% (-0.8 , 0.2)	0.615	
Your primary care physician or nurse										
Yes, definitely	1202 (81.9)	320 (82.5)	692 (84.6)	2.1% (-2.4 , 6.6)	0.349		82 (83.7)	1.2% (-7.0 , 9.4)	0.779	
Yes, somewhat	192 (13.1)	56 (14.4)	97 (11.9)	-2.6% (-6.7 , 1.6)	0.209		13 (13.3)	-1.2% (-8.7 , 6.4)	0.767	
No	27 (1.8)	‡	17 (2.1)	0.3% (-1.4 , 1.9)	0.750		‡	-0.8% (-3.2 , 1.6)	0.586	
Missing	47 (3.2)	‡	12 (1.5)	0.2% (-1.2 , 1.6)	0.806		‡	0.8% (-2.3 , 3.8)	0.577	
Experts in COVID from hospitals or public health schools										
Yes, definitely	1050 (71.5)	278 (71.6)	617 (75.4)	3.8% (-1.6 , 9.1)	0.161		70 (71.4)	-0.2% (-10.2 , 9.8)	0.965	
Yes, somewhat	304 (20.7)	92 (23.7)	147 (18.0)	-5.7% (-10.7 , -0.8)	0.019	*	21 (21.4)	-2.3% (-11.4 , 6.9)	0.633	
No	71 (4.8)	16 (4.1)	44 (5.4)	1.3% (-1.3 , 3.8)	0.349		‡	2.0% (-3.1 , 7.1)	0.395	
Missing	43 (2.9)	‡	‡	0.7% (-0.3 , 1.7)	0.248		‡	0.5% (-1.6 , 2.6)	0.569	
The governor, mayor or other government officials										
Yes, definitely	818 (55.7)	185 (47.7)	517 (63.2)	15.5% (9.6 , 21.5)	0.000	*	43 (43.9)	-3.8% (-14.8 , 7.2)	0.500	
Yes, somewhat	432 (29.4)	144 (37.1)	207 (25.3)	-11.8% (-17.5 , -6.2)	0.000	*	39 (39.8)	2.7% (-8.1 , 13.5)	0.624	
No	158 (10.8)	53 (13.7)	69 (8.4)	-5.2% (-9.1 , -1.3)	0.005	*	15 (15.3)	1.6% (-6.3 , 9.6)	0.675	
Missing	60 (4.1)	‡	25 (3.1)	1.5% (-0.2 , 3.2)	0.122		‡	-0.5% (-2.9 , 1.8)	0.696	
Your counselors or therapists										
Yes, definitely	820 (55.9)	193 (49.7)	500 (61.1)	11.4% (5.4 , 17.4)	0.000	*	51 (52.0)	2.3% (-8.8 , 13.4)	0.684	
Yes, somewhat	263 (17.9)	80 (20.6)	132 (16.1)	-4.5% (-9.2 , 0.3)	0.056		27 (27.6)	6.9% (-2.8 , 16.7)	0.139	
No	49 (3.3)	‡	31 (3.8)	1.5% (-0.5 , 3.5)	0.183		‡	0.7% (-3.0 , 4.5)	0.672	
Not Applicable	289 (19.7)	103 (26.5)	142 (17.4)	-9.2% (-14.3 , -4.1)	0.000	*	16 (16.3)	-10.2% (-18.8 , -1.7)	0.036	*
Missing	47 (3.2)	‡	13 (1.6)	0.8% (-0.4 , 2.0)	0.247		‡	0.2% (-1.9 , 2.4)	0.809	

‡Per Human Research Protocol, cell sizes of 10 or less are not reported nor are data which would allow one to deduce or calculate them. *indicates P-values < 0.05. [a]Non-Hispanic Asian and Non-Hispanic Other were excluded from this table due to small cell sizes; their responses are reflected in "All Respondents." [b]Difference in proportion between the specified

© 2023 Donelan K et al. *JAMA Health Forum.*

racial/ethnic group and non-Hispanic White with 95% confidence intervals, by response category.

© 2023 Donelan K et al. *JAMA Health Forum.*

eTable 4. Staff Reported Impacts of COVID-19 by Education Level

	Less Than College Graduation			Greater Than College Graduation				
	Total responses	Freq.	Percent	Total responses	Freq.	Percent	P-value	
**In the past year, has the COVID-19 pandemic had a very serious, somewhat serious or not serious impact on... (check one box on each row)**								
Your work or employment	674			734			0.001	*
Very serious		325	48.2%		431	58.7%		
Somewhat serious		241	35.8%		216	29.4%		
Not serious		92	13.6%		78	10.6%		
Missing		16	2.4%		9	1.2%		
Your feelings of anxiety or depression	674			734			0.324	
Very serious		189	28.0%		230	31.3%		
Somewhat serious		254	37.7%		283	38.6%		
Not serious		215	31.9%		213	29.0%		
Missing		16	2.4%		8	1.1%		
Your access to health care services when you needed them	674			734			0.592	
Very serious		184	27.3%		215	29.3%		
Somewhat serious		239	35.5%		274	37.3%		
Not serious		232	34.4%		239	32.6%		
Missing		19	2.8%		6	0.8%		
Your access to mental health services when you needed them	674			734			0.098	
Very serious		137	20.3%		128	17.4%		
Somewhat serious		173	25.7%		224	30.5%		
Not serious		340	50.4%		359	48.9%		
Missing		24	3.6%		23	3.1%		
Your contact with family or close friends	674			734			0.425	
Very serious		328	48.7%		384	52.3%		
Somewhat serious		198	29.4%		211	28.7%		
Not serious		134	19.9%		131	17.8%		
Missing		14	2.1%		8	1.1%		
Your sleep	674			734			0.831	
Very serious		166	24.6%		191	26.0%		
Somewhat serious		244	36.2%		259	35.3%		
Not serious		251	37.2%		270	36.8%		
Missing		13	1.9%		14	1.9%		
Your overall health	674			734			0.637	
Very serious		152	22.6%		162	22.1%		

© 2023 Donelan K et al. *JAMA Health Forum.*

Somewhat serious		244	36.2%		287	39.1%	
Not serious		256	38.0%		270	36.8%	
Missing		22	3.3%		15	2.0%	
The health of your family and friends	674			734			0.593
Very serious		221	32.8%		231	31.5%	
Somewhat serious		274	40.7%		322	43.9%	
Not serious		163	24.2%		173	23.6%	
Missing		16	2.4%		8	1.1%	

*indicates P-values < 0.05.

© 2023 Donelan K et al. *JAMA Health Forum.*

eTable 5. Staff Reported Needs for Support by Education Level

	Less Than College Graduation			Greater Than College Graduation			
	Total responses	Freq.	Percent	Total responses	Freq.	Percent	P-value
**Do you feel that you need extra help right now with any of the following (check one box on each row)**							
Mental or emotional health problems?	674			734			0.179
Yes		98	14.5%		125	17.0%	
No		540	80.1%		560	76.3%	
Do not know		33	4.9%		47	6.4%	
Missing		3	0.4%		2	0.3%	
Physical health problems?	674			734			0.541
Yes		87	12.9%		95	12.9%	
No		565	83.8%		609	83.0%	
Do not know		17	2.5%		26	3.5%	
Missing		5	0.7%		4	0.5%	
Coping with conflicts with other people in this home?	674			734			0.393
Yes		57	8.5%		70	9.5%	
No		584	86.6%		640	87.2%	
Do not know		25	3.7%		19	2.6%	
Missing		8	1.2%		5	0.7%	
Health and wellness? (for example, eating, exercise, smoking)	674			734			0.364
Yes		173	25.7%		215	29.3%	
No		475	70.5%		499	68.0%	
Do not know		17	2.5%		17	2.3%	
Missing		9	1.3%		3	0.4%	
Grief or loss because of things that happened during the COVID pandemic?	674			734			0.081
Yes		114	16.9%		131	17.8%	
No		530	78.6%		556	75.7%	
Do not know		23	3.4%		43	5.9%	
Missing		7	1.0%		4	0.5%	
Loneliness from being separated from family and friends in the pandemic?	674			734			0.456
Yes		138	20.5%		150	20.4%	
No		502	74.5%		542	73.8%	
Do not know		23	3.4%		35	4.8%	
Missing		11	1.6%		7	1.0%	

© 2023 Donelan K et al. *JAMA Health Forum.*

eTable 6. Staff Reported Trust in Sources of COVID-19 Information by Education Level

	Less Than College Graduation			Greater Than College Graduation			
	Total responses	Freq.	Percent	Total responses	Freq.	Percent	P-value
**For each item, indicate if you definitely or somewhat trust them to tell you the truth about COVID-19 or not.**							
Your family members	674			734			0.008
Yes, definitely		585	86.8%		602	82.0%	
Yes, somewhat		63	9.3%		109	14.9%	
No		21	3.1%		22	3.0%	
Missing		5	0.7%		1	0.1%	
Staff and directors of your group home	674			734			0.004
Yes, definitely		461	68.4%		445	60.6%	
Yes, somewhat		178	26.4%		240	32.7%	
No		26	3.9%		43	5.9%	
Missing		9	1.3%		6	0.8%	
Your primary care physician or nurse	674			734			0.288
Yes, definitely		563	83.5%		610	83.1%	
Yes, somewhat		80	11.9%		107	14.6%	
No		15	2.2%		12	1.6%	
Missing		16	2.4%		5	0.7%	
Experts in COVID from hospitals or public health schools	674			734			0.392
Yes, definitely		486	72.1%		541	73.7%	
Yes, somewhat		143	21.2%		155	21.1%	
No		38	5.6%		30	4.1%	
Missing		7	1.0%		8	1.1%	
The governor, mayor or other government officials	674			734			0.303
Yes, definitely		385	57.1%		413	56.3%	
Yes, somewhat		191	28.3%		229	31.2%	
No		82	12.2%		74	10.1%	
Missing		16	2.4%		18	2.5%	
Your counselors or therapists	674			734			0.470
Yes, definitely		393	58.3%		404	55.0%	
Yes, somewhat		114	16.9%		145	19.8%	
No		21	3.1%		27	3.7%	
NA		136	20.2%		149	20.3%	
Missing		10	1.5%		9	1.2%	

© 2023 Donelan K et al. *JAMA Health Forum.*

*indicates P-values < 0.05.

© 2023 Donelan K et al. *JAMA Health Forum.*
