# Supplementary material for: Group Home Staff Experiences With Work and Health in the COVID-19 Pandemic in Massachusetts
Source: JAMA Health Forum. 2023 Apr 7;4(4):e230445. doi: 10.1001/jamahealthforum.2023.0445 (PMC10082407; doi:10.1001/jamahealthforum.2023.0445)
Supplement: Supplement 2. — Data sharing statement [file jamahealthforum-e230445-s002.pdf]

Data Sharing Statement

Donelan. Group Home Staff Experiences With Work and Health in the COVID-19 Pandemic in Massachusetts. *JAMA Health Forum*. Published April 07, 2023.
doi:10.1001/jamahealthforum.2023.0445

Data
Data available: Yes
Data types: Deidentified participant data, Other (please specify)
Additional Information: questionnaire
How to access data: Data will be archived with authors at Massachusetts General Hospital and will be available on request from the authors after completion of research papers and required reports. The questionnaire is published in the Supplement and may be used in whole or in part with permission and appropriate citation.
When available: beginning date: 01-01-2025, end date: 01-01-2030

Supporting Documents
Document types: None

Additional Information
Who can access the data: Researchers with approved research plan
Types of analyses: specified purpose
Mechanisms of data availability: signed agreement
